# Histological subtype is the most important determinant of survival in metastatic papillary thyroid cancer

**DOI:** 10.1186/1756-6614-4-12

**Published:** 2011-07-19

**Authors:** Alexandra Chrisoulidou, Maria Boudina, Athanasios Tzemailas, Eleni Doumala, Pashalia K Iliadou, Frideriki Patakiouta, Kalliopi Pazaitou-Panayiotou

**Affiliations:** 1 Department of Endocrinology & Endocrine Oncology, Theagenio Cancer Hospital, Thessaloniki 54007, Greece; 2 Department of Nuclear Medicine, Theagenio Cancer Hospital, Thessaloniki 54007, Greece; 3 Department of Pathology, Theagenio Cancer Hospital, Thessaloniki, Greece

**Keywords:** papillary thyroid carcinoma, distant metastases

## Abstract

**Background:**

Papillary thyroid cancer (PTC) comprises the commonest type of thyroid cancer and carries the highest rate of survival. However, when metastatic disease occurs, survival is significantly affected.

**Methods:**

We aimed to identify prognostic histopathological and clinical factors that modify survival in metastatic PTC. All cases of metastatic PTC treated at our department in the last 20 years were reviewed and analyzed.

**Results:**

Histological subtype was the most important determinant of survival, as classic PTC demonstrated clearly improved survival compared to follicular subtype of PTC and other less frequently seen histological subtypes. The instant risk of death for the other histological subtypes was 4.56 times higher than the risk for the classic papillary type. Overall, a 10-year survival of 76.6% in our patients was seen.

**Conclusions:**

Patients with aggressive variants of PTC are more at risk for the development of metastatic disease. In these patients, established treatment modalities (surgery, radioiodine therapy) should be offered promptly, as well as close follow-up.

## Background

Differentiated thyroid cancer, comprising papillary and follicular thyroid cancer, generally carries a good prognosis. Papillary thyroid cancer (PTC) is the most frequent type of thyroid malignancy and its metastases are usually lymphatic [1]. The risk of distant metastases, usually to the lung, mediastinal lymph nodes and bone, is greater in follicular than in papillary carcinoma [2]. Distant metastases from PTC may occur with a frequency ranging from 1.73-8.4% in most studies [3,4]. The most common site of distant metastases from PTC is the lung followed by mediastinal lymph nodes [5]. Less often, distant metastases may appear in bones [6], central nervous system [7,8], liver [9], pericardium and pleura [10], kidney [11], pancreas [12], skin and muscle [13], gastrointestinal tract [14]. As effective treatment may not exist for many of these patients, it is of paramount importance to identify, if possible, those who are at greater risk for developing metastatic disease.

A limited number of retrospective studies have analyzed the prognostic factors, which affect clinical outcome in metastatic PTC. Prognostic factors as sex and age, tumor size, histologic type, tumor infiltration, vascular or lymphatic invasion, have been shown to affect survival in these patients [15]. However, most studies looked into prognostic factors in differentiated thyroid cancer, including both papillary and follicular thyroid cancer in the analysis. In recent years, marked differences in prognostic factors, clinicopathologic features and treatment necessitate the distinction of these two entities [4]. Even in patients with metastatic disease, the overall survival in papillary thyroid cancer is higher than in follicular cancer [16].

In this retrospective study we aimed to investigate patients with metastatic PTC only in order to delineate specific prognostic factors affecting survival in this rare entity. Within this group, we subdivided patients according to histologic subtype, and studied the clinical and histological characteristics and the received treatment modalities during a mean follow-up of 9 years. Using multivariate analysis, we estimated the factors that significantly affect survival in metastatic PTC.

## Patients and methods

We retrospectively reviewed the records of 1550 patients who had PTC. From this cohort, we identified 52 patients (29 females and 23 males) who presented with or developed (during follow up) distant metastatic disease. For all patients we collected data regarding histological features, age at diagnosis, site of distant metastases, treatment modalities and outcome of the disease. All patients were followed up at the Department of Endocrinology & Endocrine Oncology of Theagenio Cancer Hospital from 1988-2009. Patients' characteristics are shown in Table 1. Tumor, lymph nodes and metastases (TNM) staging at the time of diagnosis is shown in table 2.

**Table 1:** Characteristics of the 52 patients with metastatic PTC

	Number of patients	Number of deaths (%)
**Age (years)**		

> 19	7 (4F/3M)	1 (1M) (14.3%)
		
19-45	10 (6F/4M)	4 (3F/1M) (40.0%)
		
< 45	35 (19F/16M)	16 (6F/10M) (45.7%)

**Sex**		

Males	23	12
		
Females	29	9

**Histology**		
		
Classic papillary	24	
		
Follicular variant	20	
		
Papillary with low differentiation of the cells	6	
		
Insular	1	
		
Columnar	1	

**Size of tumor (mm)**	33 (7-80)	

**Multifocal tumors**	27 (15F/12M)	

**Bilateral tumors **	25 (13F/12M)	

**Invasion of tumor capsule**	4	

**Absence of tumor capsule**	34	

**Thyroid capsule invasion **	43	

**Extrathyroidal invasion**	38	

**Vascular infiltration**	17	

**Presence of metastases**		
At diagnosis	17	
During follow-up	35	

**Years of follow-up (years)**	8 (1-29)	

**Thyroidectomy**		
TT	46	
NTT	6	

**Total RAI dose (mCi)**	360 (100-1500)	

**Thyroglobulin at 1^st ^RAI**	121 (1.9-4000)	

Data as median (range); F: female, M: male, TT: total thyroidectomy, NTT: near total thyroidectomy.

**Table 2:** TNM staging of patients at initial diagnosis

	Ν0 (n = 20)			Ν1 (n = 32)			Total (n = 52)
	
	n		n	n		n	n
***T1***		Μ0	2		Μ0	1	
	**3**	Μ1	1	**3**	Μ1	2	**6**

**T2**		M0	1		M0	2	
	**1**	M1	0	**3**	M1	1	**4**

**T3**		M0	10		M0	9	
	**12**	M1	2	**16**	M1	7	**28**

**T4**		M0	2		M0	3	
	**4**	M1	2	**10**	M1	7	**14**

T: tumor, N: lymph nodes, M: metastases, n represents the number of patients in each category

According to AJCC Cancer Staging Manual, Sixth Edition (2002) published by Springer-Verlag, Inc., New York.

The diagnosis of distant metastases was based on whole body scan findings and elevated thyroglobulin levels and was verified by computed tomography or MRI. Whenever feasible, biopsy of the metastatic lesions confirmed the diagnosis. Upper mediastinal metastases were not considered as distant. Metastases were classified according to the site of involvement. Histological classification was in accordance with WHO classification 2004 [17].

In all patients work-up included 1) measurements of thyroglobulin, TSH and free thyroxine on suppressive therapy and 2) neck ultrasound annually. Diagnostic whole body scans and thyroglobulin after thyroxine withdrawal were performed at 9-12 months after treatment with ^131^I and when necessary thereafter. The Institutional Review Board approved the study.

### Statistical analysis

The Cox proportional hazards model was used for the assessment of possible predictors of survival. Variables with p < 0.20 in the univariate analysis were included in the multivariate analysis. Forward stepwise methodology was used for the multivariate analysis. Variables with p < 0.05 were considered statistically significant in this step. Kaplan-Meier plots were constructed in order to depict survival. PASW 18.0 (IBM-SPSS Inc., Chicago, IL) was used for data analysis. Factors considered for prediction of survival were summarised in Table 3.

**Table 3:** Factors considered for prediction of survival

Factor	Significance
Sex	0.523

Age	0.132

Histology	0.004

Multifocality	0.887

Bilaterality	0.292

Type of surgery	0.603

Size of tumor	0.392

Lymph node metastases at operation	0.263

Thyroid parenchyma invasion	0.502

Thyroid capsule invasion	0.095

Extrathyroidal invasion	0.863

Vessel invasion	0.058

Metastatic disease from diagnosis	0.180

First site of metastasis	0.874

Uptake at ablation	0.568

TSH on T4 withdrawal	0.782

Thyroglobulin on T4 withdrawal	0.799

Total 131 I mCi	0.426

Years to metastasis	0.115

## Results

Fifty-two patients (29 females and 23 males) were found to have distant metastases. Most patients were above 45 years of age (35 out of 52 patients). Twenty-two patients (42.3%) had distant metastases at the time of diagnosis (11 women and 11 men) and 30 patients (57.7%) developed metastatic disease during follow-up. Tumor size was ≥ 2 cm in 39 patients. Regional lymph node metastases were present in 30 patients. All patients had total or near total thyroidectomy. Clinical characteristics of the patients and specific characteristics of the tumors are shown in Table 1.

Twenty-four patients had classic papillary thyroid cancer, 20 patients follicular variant of papillary cancer, 1 had papillary carcinoma with insular components and 1 patient had columnar cell variant of papillary carcinoma. The remaining 6 cases exhibited papillary cancer with low differentiation of the cells (with focal follicular elements in 2 patients).

The commonest sites of metastatic disease were lung (32 patients), followed by bone (13 patients), lower mediastinum (6 patients) extracervical soft tissue (4 patients), central nervous system (4 patients), pleura (4 patients), liver (1 patient), and oeshophagus (1 patient). Multiple site metastases were found in 14 patients.

Table 3 indicates the factors affecting survival in our series: histology comprised the most significant factor in the analysis, the classic papillary type carrying significantly improved survival than follicular and other subtypes of papillary thyroid cancer. The instant risk of death for the other histological subtypes was 4.56 times higher than the risk for the classic papillary type. The other parameters tested did not affect survival significantly.

**Figure 1:**
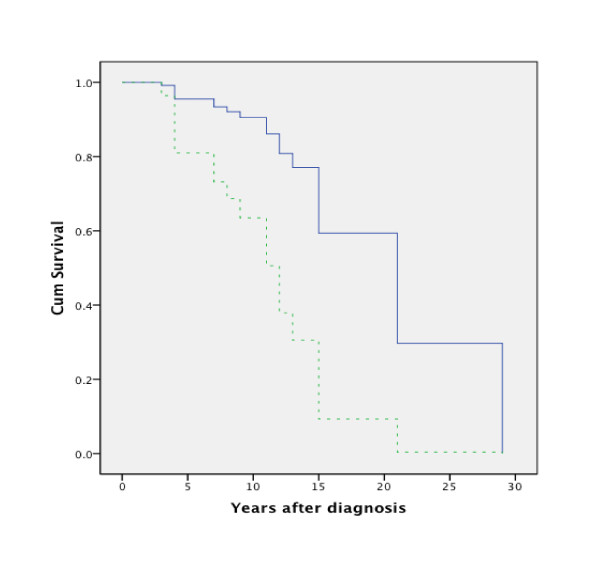
**Survival over time in patients with classic PTC**. (continuous line) and PTC variants (dotted line).

Mortality ranged according to age (table 1), with the worst outcome in patients above 45 years of age. Overall 5-year survival in metastatic papillary thyroid cancer was 88.2%, 10-year 76.6% and 15-year survival 35.8% (for 10 patients). Cumulative survival is shown in Figure 1.

## Discussion

Only a few studies so far examined specific factors affecting survival in patients with PTC, as in most studies patients with papillary and follicular thyroid cancer were included. The striking finding in this study was that histological variant plays the most significant role in patient's survival in metastatic PTC. The classic papillary type of PTC demonstrated improved prognosis compared to other papillary cancer variants.

Although papillary thyroid carcinoma carries the most favorable prognosis amongst all types of thyroid cancer, distant metastatic disease may occur with subsequent compromise in patient's survival. Even in the presence of metastatic disease, papillary thyroid cancer in our series has an overall 10-year survival of 76.6%. In general, disease-related mortality varies greatly in PTC: papillary microcarcinomas exhibit no mortality at all [18], even in the presence of distant metastases [19]. In other series with larger PTCs, 10-year survival ranged from 14-80% [20-22], with limited survival in older patients. Old age is a predictor of worse prognosis, although an exact cut off point was not uniformly identified [23]. In this study, we did not observe statistical differences in survival according to age, although we also observed an increase in mortality in patients above 45 years of age.

Previous studies indicated many factors, as capsular infiltration, extrathyroidal extension and lymph node metastases at diagnosis as unfavorable prognostic factors for persistent disease or recurrences [24]. Cervical lymph node metastases have been related to poor prognosis occurring synchronously or metachronously to diagnosis [25], although this was not seen in the present study. Extranodal invasion and perithyroidal invasion to muscles and soft tissues showed a higher incidence of distant metastases and death [26]. The presence of vessel invasion was associated, as in other reports, with poor prognosis, although in our series this association had a marginal significance (Table 2).

Although differentiated thyroid cancer is frequently seen in women, in patients with metastatic PTC the female: male ratio was 1.26:1, in accordance with the general notion that male gender adversely affects outcome in differentiated thyroid cancer. In the studied group 52% of males and 31% of females died during follow-up, however this was not significant probably due to small numbers. Histology increased nearly 5-fold the risk of death in patients with PTC variants other than the classic papillary type.

In keeping with other reports, we observed lung as the primary metastatic site, followed by bone, multiple site involvement and other, less frequently seen, affected organs. The development of rare metastases, as CNS, liver and extracervical soft tissue metastases, indicates the importance of close clinical follow-up in these patients.

In conclusion, the histological subtype in metastatic PTC determines survival in a significant way. As a result, patients with follicular and other uncommon subtypes of PTC that present with or develop distant metastatic disease should be vigorously treated with surgery, where appropriate, radioiodine therapy and should also be considered for inclusion to studies concerning new therapies, like tyrosine kinase inhibitors.

## Competing interests

The authors declare that they have no competing interests.

## Authors' contributions

KPP conceived and coordinated the study. MB, PKI, ED gathered the data. MB and AT prepared the database and inserted the relevant data. FP examined the histological material. AC did the statistical analysis and drafted the tables. KPP and AC wrote the manuscript. All authors read and approved the final manuscript.
